# Buyang Huanwu Tang (Boyang Hwano Tang) for the treatment of post-stroke fatigue: Protocol for a systematic review of randomized controlled trials

**DOI:** 10.1097/MD.0000000000017116

**Published:** 2019-09-13

**Authors:** Chul Jin, Seung-Yeon Cho, Seong-Uk Park, Woo-Sang Jung, Sang-Kwan Moon, Jung-Mi Park, Chang-Nam Ko, Ki-Ho Cho, Seungwon Kwon

**Affiliations:** Department of Cardiology and Neurology, College of Korean Medicine, Kyung Hee University, Seoul, Korea (Republic of).

**Keywords:** Buyang Huanwu Tang, post-stroke fatigue, randomized controlled trial, systematic review

## Abstract

**Background:**

Buyang Huanwu Tang (BHT) is a well-known herbal complex used for stroke treatment and has been used mainly during post-stroke rehabilitation in East Asia. In this review, we aim to evaluate the efficacy and safety of BHT as a treatment for post-stroke fatigue (PSF).

**Methods:**

Eight databases will be searched for relevant studies from inception to the present date. We will include randomized controlled trials (RCTs), which assess the effect and safety of BHT for the treatment of PSF. The methodological qualities, including the risk of bias (RoB), will be evaluated using the Cochrane RoB assessment tool. After screening the studies, a meta-analysis of the RCTs will be done.

**Results:**

This study will provide high-quality synthesis of current evidence of BHT for PSF.

**Conclusion:**

The conclusion of our systematic review will provide evidence to judge whether BHT is an effective intervention for patients with PSF.

**Ethics and dissemination:**

Ethical approval is not required, as this study is based on the review of published research. This review will be published in a peer-reviewed journal and disseminated both electronically and in print.

**Trial registration number:**

PROSPERO CRD42019130178.

## 1 Introduction

Stroke is defined as a state in which partial or total rapid development of cerebral dysfunction, caused solely by cerebrovascular disease, lasts for a considerable period of time.^[1]^ Most stroke patients complain of motor or sensory disturbances on one side of the body. These symptoms are known to decrease with regular exercise.^[2]^ However, the opportunity for recovery is limited: recovery of neurological deficits after stroke is difficult after an average of 3 to 6 months.^[2]^

Several studies have reported that post-stroke fatigue (PSF) occurs in 30% to 72% of stroke patients.^[3–9]^ Fatigue after stroke will adversely affect rehabilitation training. According to an article by Nadarajah and Goh^[10]^ in 2015, PSF is associated with physical deconditioning, reduced self-efficacy for physical performance, poor rehabilitation participation and outcome, reduced social participation, poor quality of life, functional limitation, and increased mortality. As such, PSF has an adverse effect on the recovery of stroke patients. Although various therapies have been used, there is no established treatment for PSF. According to the 2009 Cochrane Review,^[11]^ fluoxetine, tirilazad, and self-management programs did not show a significant improvement when compared to the control treatment.^[11]^

Several thousand years ago, various herbal complexes have been used to treat stroke in East Asia. Among them, Buyang Huanwu Tang (BHT, Boyang-Hwano-Tang in Korean, Hokango-to in Japanese), a well-known herbal complex that helps treat stroke, has been used mainly during post-stroke rehabilitation.^[12]^ BHT consists of the following 7 herbs: Radix Astragali seu Hedysari, Radix Angelica Sinensis, Radix Paeoniae Rubra, Rhizoma Ligustici Chuanxiong, Flos Carthami, Semen Persicae, and Pheretima. Traditionally, BHT has been used to enhance the power of Qi and blood circulation, thereby improving blood stasis.^[12]^ According to recent studies, BHT has shown neuroprotective effects in various experimental models,^[13–15]^ the specific mechanism is as follows: promotion of growth and differentiation of neural cells,^[13]^ inhibition of neuronal cell apoptosis,^[13]^ Ca^2+^ overload reduction,^[14]^ and inflammatory response.^[15]^

Emerging randomized controlled trials (RCTs) have also continuously reported the efficacy and safety of BHT for stroke treatment. A systematic review and meta-analysis of these studies was performed to evaluate the efficacy and safety of BHT for the treatment of acute ischemic stroke,^[16,17]^ but no systematic review or meta-analysis has been performed to evaluate the clinical efficacy and safety of using BHT for PSF treatment.

The aims of this study are as follows:

(1) To assess whether PSF therapy using BHT is more effective and safe than conventional western medicine therapies or placebo
(2) To assess whether PSF therapy using a combination of BHT and conventional western medicine therapies is safer and more effective than conventional western medicine therapies alone.

## 2 Methods

### 2.1 Study registration

The current protocol report adheres to the Preferred Reporting Items for Systematic Reviews and Meta-Analysis (PRISMA) Protocols.^[18]^ The protocol for this systematic review has been registered in PROSPERO 2019 under number CRD42019130178**.**

### 2.2 Eligible criteria for study selection

#### 2.2.1 Types of studies

Only RCTs and quasi-RCTs investigating BHT for PSF treatment will be included in this study, without publication or language restriction. Non-RCTs, case reports, case series, uncontrolled trials, and laboratory studies will be excluded, as will trials that fail to provide detailed results.

#### 2.2.2 Types of participants

The eligible participants will be patients with PSF. Qualified clinical diagnosis methods, including the Fatigue Assessment Scale (FAS)^[19]^ and the Fatigue Severity Scale (FSS),^[19]^ and subjective fatigue symptoms will be used for the diagnosis of PSF. There will be no restrictions based on sex, ethnicity, symptom severity, disease duration, and clinical setting. Patients with other conditions that can, however, cause fatigue such as cancer, chronic kidney diseases, and liver cirrhosis will be excluded.

#### 2.2.3 Types of interventions

We will include studies using BHT and modified (herbs added) BHT as experimental interventions, with no limitations on dosage, frequency, duration of treatment, and formulation (eg, decoctions, extracts, tablets, capsules, and powders, etc).This study will only include oral administration of BHT or modified BHT. Intravenous or acupuncture points injections will be excluded. The control interventions will include no treatment, placebo, or conventional western medicine therapies. Both treatment with BHT or modified BHT alone and adjunctive treatment with conventional therapies will be considered acceptable if BHT or modified BHT is applied only to the intervention group and conventional treatment is provided equally to both the intervention and control group. We will exclude studies comparing other traditional Chinese medicine therapies (including traditional Korean medicine and Japanese Kampo Medicine) such as those using different types of herbal medicines, acupuncture treatments, or moxibustions.

#### 2.2.4 Types of outcome measures

For the primary outcome, we will assess the FAS scores to evaluate the severity of fatigue. For secondary outcomes, we will include other parameters such as FSS scores, total clinical effective rate (percentage of patients whose fatigue symptom was improved), inflammatory cytokine levels. We will also investigate the number and severity of adverse events.

### 2.3 Search methods for the identification of studies

#### 2.3.1 Electronic searches

The following databases will be searched from inception to February 2019: MEDLINE (via PubMed), the Cochrane Central Register of Controlled Trials, Scopus, Citation Information by Nii, China National Knowledge Infrastructure Database, Oriental Medicine Advanced Searching Integrated System, National Digital Science Library, and Korean Traditional Knowledge Portal.

The terms to be used for searching include (Buyang Huanwu OR Boyang Hwano OR Hoyangkango) AND (Poststroke fatigue OR PSF OR stroke fatigue OR fatigue after stroke OR [cerebrovascular accident AND fatigue] OR [CVA AND fatigue] OR [stroke AND fatigue]).

The specific search strategies (eg, for PubMed) are as follows:

1. ((Buyang Huanwu [Title/Abstract]) OR Boyang Hwano [Title/Abstract]) OR Hoyangkango [Title/Abstract].
2. ((((((Poststroke fatigue [Title/Abstract]) OR PSF [Title/Abstract]) OR stroke fatigue [Title/Abstract]) OR fatigue after stroke [Title/Abstract]) OR (cerebrovascular accident AND fatigue) [Title/Abstract]) OR (CVA AND fatigue) [Title/Abstract]) OR (stroke AND fatigue) [Title/Abstract].
3. (((random [Text Word] OR randomized [Text Word]) OR control [Text Word]) OR controlled [Text Word]) OR trial [Text Word] AND “humans” [MeSH Terms]
4. #1AND#2AND#3.

We will make relative modifications in accordance with the requirements, and an equivalent translation of the search terms will be adopted to ensure that similar search terms are used in all databases.

#### 2.3.2 Search for other resources

A manual search will also be done to search the reference lists of the relevant articles.

### 2.4 Data collection and analysis

#### 2.4.1 Study selection

**Figure 1:**
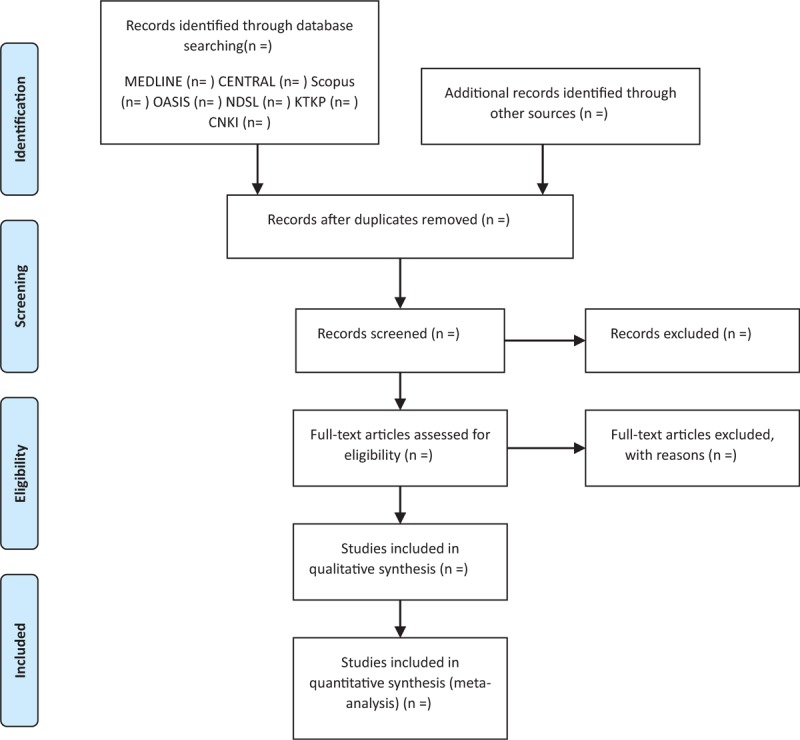
A Preferred Reporting Items for Systematic Reviews and Meta-Analysis (PRISMA) flow diagram of literature screening and selection processes.

Two reviewers (SK and CJ) who have been trained on the process and purpose of selection will independently review the titles, abstracts, and manuscripts of the studies and screen them for eligibility for inclusion in the analysis. After removing duplicates, full-texts will be reviewed. All studies, identified by both electronic and manual searches will be uploaded to EndNote X7 (Clarivate Analytics), and the reasons for excluding studies will be recorded and shown in a PRISMA flowchart as shown in Figure 1. All disagreements will be resolved by a consensus and discussion between the 2 reviewers.

#### 2.4.2 Data extraction and management

Two review writers (SK and CJ) will independently extract the data and fill out the standard data extraction form, which includes study information such as the first author, publication year, language, research design, sample size, characteristics of participants (eg, age, sex), details of randomization, blinding, interventions, treatment period, outcome measures, primary outcome, secondary outcome, statistical method used, etc. If there are disagreements, another review writer (W-SJ) will be called in to help make the decision.

#### 2.4.3 Assessment of bias risk and quality of included studies

Two reviewers (SK and CJ) will assess the risk of bias (RoB) based on the Cochrane Collaboration's tool,^[20]^ which includes references to the following: random sequence generation (selection bias), allocation concealment (selection bias), blinding of participants and personnel (performance bias), blinding of outcome assessment (detection bias), incomplete outcome data (attribution bias), selective reporting (reporting bias), and other bias. The assessment results will be shown as 1 of the 3 categories: low, unclear, and high.

#### 2.4.4 Measurement of treatment effect

For continuous data, the pooled results will be presented as the mean difference (MD) or standardized MD with 95% confidence intervals (CIs). For dichotomous data, the pooled results will be presented as a risk ratio with 95% CIs.

#### 2.4.5 Managing missing data

It there is any missing, insufficient, or unclear data, we will contact the corresponding author and gather the relevant information. If the information cannot be obtained, only the remaining available information will be analyzed, and it will be discussed.

#### 2.4.6 Assessment of heterogeneity

We will perform the *I*^2^ test to evaluate the statistical heterogeneity. If the *I*^2^ is >50%, statistical heterogeneity will be significantly considered.

#### 2.4.7 Data synthesis

We will use the Review Manager program (V.5.3.5 Copenhagen: The Nordic Cochrane Centre, The Cochrane Collaboration, 2014) to perform statistical analysis. If the *I*^2^ ≤ 50%, the fixed-effect model will be employed to evaluate the outcome data. Otherwise, the random effects model will be applied. The studies will be synthesized according to the type of intervention and/or control as follows:

1. BHT vs no treatment,
2. BHT vs placebo control,
3. BHT vs conventional western medicine,
4. BHT + conventional western medicine combined therapy vs only conventional western medicine therapy.

Modified BHT will be included in the BHT group as described in the “intervention section.”

The heterogeneity levels will be assessed in the included literature, and, if enough studies are available to investigate the causes of heterogeneity and its criteria, the groups indicated below (“Analysis of subgroups or subsets section”) will be assessed. We will use Grading of Recommendations Assessment, Development and Evaluation pro software from Cochrane Systematic Reviews to create a Summary of Findings table.

#### 2.4.8 Subgroup analysis

If enough studies are available to investigate the cause of heterogeneity and its criteria, the following will be assessed: the formation of the BHT such as granules or decoctions, the number and types of additional herbs, the accompanying diseases such as hypertension, diabetes, dyslipidemia, depression, and impaired cognitive function, the types of control group (eg, antidepressants, cerebral function improving agents), and treatment period.

#### 2.4.9 Sensitivity analysis

We will perform a sensitivity analysis to verify the robustness of the study results. This will be done by assessing the impact of sample size, high RoB, missing data, and selected models. Following the analyses, if the quality of the studies is judged to be low, these studies will be removed to ensure the robustness of the results.

#### 2.4.10 Ethics and dissemination

Formal ethical approval is not required in this protocol. We will collect and analyze data based on published studies, and since no patients are directly or specifically assessed in this study, individual privacy will not be a concern. The results of this review will be disseminated to peer-reviewed journals or presented at a relevant conference.

## 3 Discussion

PSF has a negative impact on rehabilitation training of stroke patients.^[10]^ PSF not only limits the physical ability of stroke patients but also interferes with their rehabilitation therapy by delaying recovery from stroke and possibly causing complications. As a result, the patient's quality of life is continuously degraded. However, there is no definite intervention to treat PSF.^[11]^

According to a previous study, Qi deficiency, one of the pattern identification types in traditional East Asian medicine, is associated with fatigue in patients with breast cancer.^[21]^ BHT has traditionally been used for the treatment of blood stasis in the background of Qi deficiency and has been used for the improvement of hemiparesis in patients with chronic stroke.^[12]^ In addition, BHT has a neuroprotective effect and is expected to help the recovery of stroke patients when used in addition to conventional therapies.^[13–17]^ Thus, BHT is likely to be a new alternative to improve fatigue which may have been a problem for the rehabilitation of stroke patients.

The present review will be conducted to assess the efficacy and safety of using BHT to treat PSF, so as to establish management strategies that benefit and reduce the burden of practitioners, patients, and their families.

## Author contributions

**Conceptualization:** Seungwon Kwon.

**Data curation:** Chul Jin, Seung-Yeon Cho, Woo-Sang Jung.

**Formal analysis:** Seong-Uk Park, Woo-Sang Jung, Sang-Kwan Moon, Jung-Mi Park.

**Funding acquisition:** Seungwon Kwon.

**Project administration:** Seungwon Kwon.

**Writing – original draft:** Chul Jin, Seungwon Kwon.

**Writing – review and editing:** Chang-Nam Ko, Ki-Ho Cho, Seungwon Kwon.
